# Supplementary material for: The Relevance of Pharmacokinetic Biomarkers in Response to Methadone Treatment: A Systematic Review
Source: Pharmaceuticals (Basel). 2025 Apr 25;18(5):623. doi: 10.3390/ph18050623 (PMC12115004; doi:10.3390/ph18050623)
Supplement: Supplementary file 1 [file pharmaceuticals-18-00623-s001.zip › Supplementary Tables.pdf]

**Supplementary Table S1. Search strategies for each database.** The search strategy for this systematic review was modified when necessary. Automatic initial filters that were common for all databases were used (year of publication (2000-2024, both included), and language (English)).

| Database | Search strategy | Results |
|---|---|---|
| **PsycINFO[1]** | (methadone) AND (metabolism) AND (gene* OR polymorphism* OR biomarker*) | **22** |
| **PubMed[2]** | (methadone[Title/Abstract] AND metabolism[Title/Abstract] AND (gene*[Title/Abstract] OR polymorphism*[Title/Abstract] OR biomarker*[Title/Abstract])) | **87** |
| **Scopus[3]** | TITLE-ABS-KEY ( ( methadone ) AND ( metabolism ) AND ( gene* OR polymorphism* OR biomarker* ) ) | **732** |
| **Web of Science[4]** | (TI=(methadone) OR AB=(methadone) OR KP=(methadone)) AND (TI=(metabolism) OR AB=(metabolism) OR KP=(metabolism)) AND (TI=(gene*) OR AB=(gene*) OR KP=(gene*) OR TI=(polymorphism*) OR AB=(polymorphism*) OR KP=(polymorphism*) OR TI=(biomarker*) OR AB=(biomarker*) OR KP=(biomarker*)) | **203** |

[1]PsycINFO: Access is provided through the library of the University of Santiago de Compostela (https://www.proquest.com/psycinfo/index?accountid=17253&parentSessionId=x0feycV5CrvA1o2Jve%2BCRsj7eswO1TpvPGfquf6EuBc%3D)

[2] PubMed Central®: https://pubmed.ncbi.nlm.nih.gov/

[3] Scopus: https://www.scopus.com/search/form.uri?display=basic#basic

[4] Web of Science: https://www.webofscience.com/wos/woscc/basic-search

**Supplementary Table S2. Risk of bias assessment of cross-sectional studies.** The NHLBI Study Quality Assessment Tool for Observational Cohort and Cross-Sectional Studies was used for detecting potential flaws in each of the ten final included articles of this type.

| CROSS-SECTIONAL STUDIES | Eap et al, 2001 | Crettol et al, 2005 | Eap et al, 2006 | Lötsch et al, 2006 | Crettol et al, 2006 | Pérez de los Cobos et al, 2006 | Fonseca et al, 2011 | Dobrinas et al, 2013 | Mouly et al, 2014 | Carlquist et al, 2015 |
|---|---|---|---|---|---|---|---|---|---|---|
| 1. Was the research question or objective in this paper clearly stated? | ✓ | ✓ | ✓ | ✓ | ✓ | ✓ | ✓ | ✓ | ✓ | ✓ |
| 2. Was the study population clearly specified and defined? | ✗ | ✓ | ✗ | ✗ | ✓ | ✗ | ✗ | ✓ | ✓ | ✓ |
| 3. Was the participation rate of eligible persons at least 50%? | ✓ | ✓ | ✓ | ✓ | ✓ | ✓ | ✓ | ✓ | ✓ | ✗ |
| 4. Were all the subjects selected or recruited from the same or similar populations (including the same time period)? Were inclusion and exclusion criteria for being in the study prespecified and applied uniformly to all participants? | ✓ | ✗ | CD | ✗ | ✗ | ✓ | ✓ | ✓ | ✗ | ✓ |
| 5. Was a sample size justification, power description, or variance and effect estimates provided? | ✓ | ✗ | ✗ | ✗ | ✗ | ✗ | ✗ | ✗ | ✗ | ✗ |
| 6. For the analyses in this paper, were the exposure(s) of interest measured prior to the outcome(s) being measured? | ✓ | ✓ | ✓ | ✓ | ✓ | ✗ | ✓ | ✓ | ✓ | ✓ |
| 7. Was the timeframe sufficient so that one could reasonably expect to see an association between exposure and outcome if it existed? | ✓ | ✓ | ✓ | ✓ | ✓ | ✓ | ✓ | ✓ | ✓ | ✓ |
| 8. For exposures that can vary in amount or level, did the study examine different levels of the exposure as related to the outcome (e.g., categories of exposure, or exposure measured as continuous variable)? | ✓ | ✓ | ✓ | ✓ | ✓ | NA | ✓ | ✓ | ✓ | ✓ |
| 9. Were the exposure measures (independent variables) clearly defined, valid, reliable, and implemented consistently across all study participants? | ✓ | ✓ | ✓ | ✓ | ✓ | ✓ | ✓ | ✓ | ✓ | ✓ |
| 10. Was the exposure(s) assessed more than once over time? | ✗ | ✗ | ✗ | ✗ | ✗ | ✗ | ✗ | ✗ | ✗ | ✗ |
| 11. Were the outcome measures (dependent variables) clearly defined, valid, reliable, and implemented consistently across all study participants? | ✓ | ✓ | ✓ | ✓ | ✓ | ✓ | ✓ | ✓ | ✓ | ✓ |
| 12. Were the outcome assessors blinded to the exposure status of participants? | NR | NR | ✓ | NR | NR | NR | NR | NR | NR | NR |
| 13. Was loss to follow-up after baseline 20% or less? | ✓ | ✓ | ✓ | ✓ | ✓ | ✓ | ✓ | ✓ | ✓ | ✗ |
| 14. Were key potential confounding variables measured and adjusted statistically for their impact on the relationship between exposure(s) and outcome(s)? | ✗ | ✓ | ✓ | ✓ | ✓ | ✓ | ✓ | ✓ | ✓ | ✓ |

CD: cannot determine; NA: not applicable; NR: not reported.

**Supplementary Table S3. Risk of bias assessment of case-control studies.** The NHLBI Study Quality Assessment of Case-Control Studies was used for detecting potential flaws in each of the four final included articles of this type

| | Bunten et al, 2010 | Christoffersen et al, 2016 | Ahmad et al, 2017 | Iwersen-Bergmann et al, 2021 | |
|---|---|---|---|---|---|
| CASE-CONTROL STUDIES | ✓ | ✓ | ✓ | ✓ | **1. Was the research question or objective in this paper clearly stated and appropriate?** |
| | ✓ | ✓ | ✓ | ✓ | **2. Was the study population clearly specified and defined?** |
| | ✓ | ✗ | ✗ | ✗ | **3. Did the authors include a sample size justification?** |
| | ✓ | ✓ | ✗ | ✗ | **4. Were controls selected or recruited from the same or similar population that gave rise to the cases (including the same timeframe)?** |
| | NR | ✓ | ✓ | ✗ | **5. Were the definitions, inclusion and exclusion criteria, algorithms or processes used to identify or select cases and controls valid, reliable, and implemented consistently across all study participants?** |
| | ✓ | ✓ | ✓ | ✗ | **6. Were the cases clearly defined and differentiated from controls?** |
| | ✓ | ✓ | ✓ | CD | **7. If less than 100 percent of eligible cases and/or controls were selected for the study, were the cases and/or controls randomly selected from those eligible?** |
| | NR | NR | NR | NR | **8. Was there use of concurrent controls?** |
| | ✓ | ✓ | ✓ | ✓ | **9. Were the investigators able to confirm that the exposure/risk occurred prior to the development of the condition or event that defined a participant as a case?** |
| | ✓ | ✓ | ✓ | ✓ | **10. Were the measures of exposure/risk clearly defined, valid, reliable, and implemented consistently (including the same time period) across all study participants?** |
| | NA | NA | NA | NA | **11. Were the assessors of exposure/risk blinded to the case or control status of participants?** |
| | CD | ✓ | ✗ | ✗ | **12. Were key potential confounding variables measured and adjusted statistically in the analyses? If matching was used, did the investigators account for matching during study analysis?** |

CD: cannot determine; NA: not applicable; NR: not reported.
